# The PpMYC2–PpEBF1–PpJAZ1 module mediates the crosstalk between jasmonate and ethylene signaling in peach disease resistance

**DOI:** 10.1093/hr/uhag207

**Published:** 2026-06-01

**Authors:** Di Ai, Lei Zhao, Yu-Jie Jiang, Lan-Xi Sha, Chao-Xi Luo, Yuepeng Han, Jian-Ping An

**Affiliations:** State Key Laboratory of Plant Diversity and Specialty Crops, Wuhan Botanical Garden, Chinese Academy of Sciences, Wuhan 430074, China; University of Chinese Academy of Sciences, Beijing 100049, China; State Key Laboratory of Plant Diversity and Specialty Crops, Wuhan Botanical Garden, Chinese Academy of Sciences, Wuhan 430074, China; State Key Laboratory of Plant Diversity and Specialty Crops, Wuhan Botanical Garden, Chinese Academy of Sciences, Wuhan 430074, China; University of Chinese Academy of Sciences, Beijing 100049, China; State Key Laboratory of Plant Diversity and Specialty Crops, Wuhan Botanical Garden, Chinese Academy of Sciences, Wuhan 430074, China; University of Chinese Academy of Sciences, Beijing 100049, China; College of Plant Science and Technology, Huazhong Agricultural University, Wuhan 430070, China; State Key Laboratory of Plant Diversity and Specialty Crops, Wuhan Botanical Garden, Chinese Academy of Sciences, Wuhan 430074, China; State Key Laboratory of Plant Diversity and Specialty Crops, Wuhan Botanical Garden, Chinese Academy of Sciences, Wuhan 430074, China

Dear Editor,

Bacterial spot (BS) disease, caused by *Xanthomonas arboricola* pv. *pruni*, leads to leaf perforation, defoliation, weakened tree vigor, and reduced yield, thereby threatening the sustainability of the peach industry [1]. However, current control strategies predominantly rely on chemical pesticides, which face multiple challenges, including environmental safety concerns and the emergence of pesticide resistance. Therefore, exploring inherent resistance and breeding disease-resistant varieties have become fundamental strategies, highlighting the urgent need to elucidate the molecular immune mechanisms underlying disease resistance in peach.

Jasmonic acid (JA) and its derivatives constitute a major class of lipid-derived phytohormones extensively present across the plant. Beyond their critical role as signaling molecules in regulating plant growth and development, JAs function as central mediators in plant adaptation to both biotic and abiotic stresses. Activation of the JA signaling pathway represents a pivotal molecular switch that orchestrates plant defense responses against diverse threats, including insect herbivory, pathogen invasion, and adaptation to environmental extremes such as drought and low temperature stress [1, 2]. In *Arabidopsis thaliana*, the biosynthesis of JA predominantly takes place through two distinct metabolic routes: the octadecanoid pathway, initiated from linolenic acid, and the hexadecanoic pathway, which utilizes hexadecatrienoic acid as a precursor. Under basal conditions, JASMONATE ZIM-DOMAIN (JAZ) proteins act as transcriptional repressors by sequestering and inhibiting transcription factors such as those in the MYC family. Upon perception of stress and the consequent elevation of endogenous JA levels, the SCF^COI1^ complex facilitates the ubiquitination and proteasomal degradation of JAZ proteins. This process liberates MYC and other related transcription factors, leading to the transcriptional activation of a wide spectrum of downstream defense-related genes and the initiation of coordinated defense responses [3].

The stability of JAZ proteins is regulated by the CORONATINE INSENSITIVE 1 (COI1) through the 26S proteasomal degradation pathway. In addition, a recent study reported that the RING-domain E3 ubiquitin ligase SEVEN IN ABSENTIA 11 also regulates JAZ stability through the proteasomal pathway [3]. Despite extensive research on the JA signaling network, the molecular mechanisms by which other regulatory factors interact with JAZ proteins to modulate their stability, as well as the mechanism through which JA regulates defense against bacterial spot disease in peach, remain to be further elucidated.

**Figure 1:**
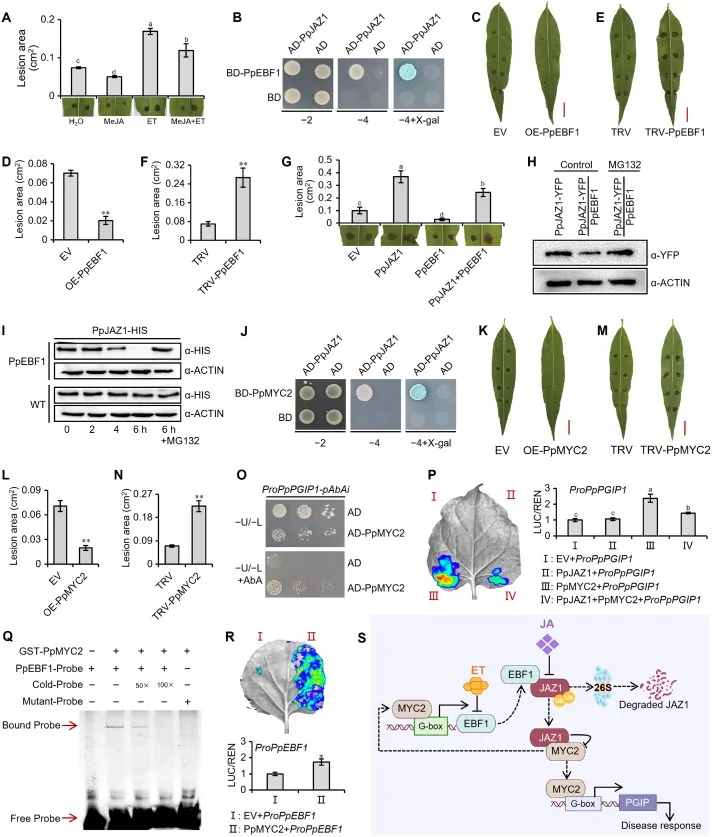
The PpMYC2–PpEBF1–PpJAZ1 module regulates disease resistance. (A) Leaf phenotypes and disease lesion areas at 7 days post-inoculation (dpi). MeJA, 50 μΜ MeJA; ET, 50 μM 1-aminocyclopropane-1-carboxylic acid (ACC); ET + MeJA, a combination of 50 μΜ MeJA and 50 μM ACC. Following pretreatment, leaves of different genotypes were inoculated with Xap and incubated in the dark at 28°C. Disease severity was assessed by measuring lesion diameter. The experiment was independently repeated three times with similar results, with each replicate containing 10 peach leaves. A representative image is shown. Error bars represent standard deviations (SD). Data are presented as mean ± SD. Different letters on top of bars indicate statistical significance at the same time point and *P* < 0.05 based on one-way ANOVA followed by Tukey’s test. (B) Y2H assay showing the physical interaction between PpEBF1 and PpJAZ1. −2, selective medium lacking tryptophan and leucine; −4, selective medium lacking tryptophan, leucine, histidine, and adenine; X-gal, 5-bromo-4-chloro-3-indolyl-β-D-galactopyranoside. (C)The phenotype and (D) lesion area of peach leaves expressing the *PpEBF1* overexpression vector (OE-PpEBF1) after inoculation. EV, the empty vector. Error bars represent SD. Data are presented as mean ± SD. Asterisks represent significant differences based on Student’s *t*-test: ^**^, *P* < 0.01. (E) The phenotype and (F) lesion area of *PpEBF1*-silenced peach leaves (TRV-PpEBF1). TRV, empty pTRV2 vector. Error bars represent SD. Data are presented as mean ± SD. Asterisks represent significant differences based on Student’s *t*-test: ^**^, *P* < 0.01. (G) Leaf pathogen inoculation assay showing the regulatory roles of *PpJAZ1* and *PpEBF1* in disease response and the effect of PpEBF1 on the disease regulatory function of *PpJAZ1*. EV, empty vector control; PpJAZ1, overexpression of *PpJAZ1*; *PpEBF1*, overexpression of *PpEBF1*; *PpJAZ1/PpEBF1*, simultaneous overexpression of *PpJAZ1* and *PpEBF1*. Error bars represent SD. Data are presented as mean ± SD. Different letters on top of bars indicate statistical significance at the same time point and *P* < 0.05 based on one-way ANOVA followed by Tukey’s test. (H) Effect of *PpEBF1* on regulation on *PpJAZ1* degradation. PpJAZ1-YFP was immunoprecipitated from *PpJAZ1* and *PpJAZ1/PpEBF1* transgenic tobacco leaves with or without 100 μM MG132 treatment. (I) *In vitro* cell-free protein degradation assay. Total proteins were extracted from WT and *PpEBF1* transiently transformed peach callus and treated with or without 100 μM proteasome inhibitor MG132, and then incubated with purified PpJAZ1-HIS protein. (J) Y2H assay showing the physical interaction between PpMYC2 and PpJAZ1. (K) The phenotype and (L) lesion area of peach leaves expressing the *PMYC2* overexpression vector (OE-PpMYC2) after inoculation. EV, empty vector. Error bars represent SD. Data are presented as mean ± SD. Asterisks represent significant differences based on Student’s *t*-test: ^**^, *P* < 0.01. (M) The phenotype and (N) lesion area of *PpMYC2*-silenced peach leaves. TRV, empty pTRV2 vector. Error bars represent SD. Data are presented as mean ± SD. Asterisks represent significant differences based on Student’s *t*-test: ^**^, *P* < 0.01. (O) Analysis of the binding of *PpMYC2* to the *PpPGIP1* promoter using Y1H assays. −U/−L, selective medium lacking uracil and leucine; −U/−L, selective medium lacking uracil and leucine with *Aureobasidin* A (600 ng/ml). The empty AD vector was used as a negative control. (P) Dual-LUC assay in *N. benthamiana* leaves was used to investigate the synergistic roles of *PpJAZ1* and *PpMYC2* in transcriptional regulation of *PpPGIP1*. The experiment was repeated three times with similar results, and each replicate included 3–4 *N. benthamiana* leaves. A representative image is shown. Error bars represent SD. Data are presented as mean ± SD. The empty vector value was used as the reference and set to 1. Different letters on top of bars indicate statistical significance at the same time point and *P* < 0.05 based on one-way ANOVA followed by Tukey’s test. (Q) EMSA showing the binding of PpMYC2 to the *ProPpEBF1* promoter in *vitro*. ‘−’ indicates no protein or probe added; ‘+’ indicates protein or probe added; ‘50×,’ ‘100×’ represent increasing concentrations of competitor probe. Mutant represents the replacement of the CACATG sequence in the *PpEBF1* promoter with AAAAAA. (R) Dual-LUC assay in *N. benthamiana* leaves was used to investigate the synergistic roles of *PpMYC2* in transcriptional regulation of *PpEBF1*. Error bars represent SD. Data are presented as mean ± SD. The empty vector value was used as the reference and set to 1. Asterisks represent significant differences based on Student’s *t*-test: ^*^, *P* < 0.05. (S) A working model for ET and JA signaling integration in disease defense via the PpMYC2–PpEBF1–PpJAZ1 module.

Plants are continually exposed to various pathogenic microorganisms and herbivorous insects in their natural environment. To cope with single or multiple attacks, phytohormone signaling pathways form complex crosstalk networks through synergistic or antagonistic interactions, enabling precise regulation of defense responses [1, 2, 4]. The JA signaling pathway, as a key component of this network, operates through the repression of the transcription factor MYC2 by JAZ proteins, and participates in disease resistance through interactions with ethylene (ET), salicylic acid, and abscisic acid signaling pathways. For example, ET not only plays a crucial role in plant defense but also exhibits substantial overlap with downstream JA-responsive genes, particularly in regulating members of the ETHYLENE RESPONSIVE FACTOR transcription factor family. These two pathways often act synergistically to enhance plant resistance against pathogens [2]. However, JA–ET interactions are not always cooperative; under specific conditions, antagonism may also occur. For instance, the core ET-responsive factors ETHYLENE-INSENSITIVE 3 (EIN3)/EIN3-LIKE 1 (EIL1) interfere with MYC2 function, thereby compromising plant defense against herbivores [4]. Currently, the detailed molecular mechanisms underlying the crosstalk between JA and ET pathways remain incompletely understood. In this study, we focus on the ET-mediated initiation and amplification of JA signaling, aiming to elucidate the molecular regulatory mechanisms in peach defense responses.

We found that methyl jasmonate (MeJA) treatment significantly reduced the lesion area caused by pathogen inoculation, whereas ET treatment aggravated the disease symptoms (Fig. 1A). Interestingly, exogenous MeJA application effectively inhibited ET-induced disease progression (Fig. 1A, Supplementary Fig. S1), suggesting an antagonistic interaction between JA and ET signaling pathways. JA and ET are often reported to act synergistically in plant defense. However, previous research has shown that EIN3/EIL1 can impair MYC2 function, thereby reducing plant resistance to insects [4]. The intricate interplay between JA and ET not only equips plants with a robust and adaptable immune system but can also be exploited by pathogens to attenuate host defenses. PpJAZ1 was identified to negatively regulate peach resistance against BS disease [1]. Yeast two-hybrid (Y2H) assay revealed a direct interaction between PpEBF1, a key regulator in the ET signaling pathway, and PpJAZ1 (Fig. 1B). This interaction was further validated through experiments such as bimolecular fluorescence complementation (BiFC) and split luciferase complementation (LCI) assays (Supplementary Fig. S2). Functional validation showed that transient overexpression of *PpEBF1* in peach leaves enhanced disease resistance (Fig. 1C and D, Supplementary Fig. S3A), while silencing of *PpEBF1* exacerbated the disease (Fig. 1E and F, Supplementary Fig. S3B). The robustness of this phenotype was further confirmed in peach fruits (Supplementary Fig. S4). These results indicate that *PpEBF1* positively regulates disease resistance.

Notably, co-expression of *PpEBF1* and *PpJAZ1* significantly alleviated the susceptible phenotype caused by *PpJAZ1* overexpression (Fig. 1G), This suggests that PpEBF1 might function by modulating PpJAZ1 protein stability to enhance disease resistance. To explore the impact of PpEBF1 on PpJAZ1 protein stability, an *in vivo* protein level assay was performed and found that overexpression of *PpEBF1* significantly reduced the accumulation of PpJAZ1 protein (Fig. 1H). Meanwhile, the proteasome inhibitor MG132 completely blocked PpEBF1-mediated degradation of PpJAZ1, indicating that PpEBF1 mediates the degradation of PpJAZ1 through the ubiquitin–proteasome pathway. We further compared the ubiquitination levels of HA-tagged PpJAZ1 protein immunoprecipitated from peach callus expressing *PpJAZ1* alone or co-expressing *PpJAZ1* and *PpEBF1*. The results showed that overexpression of *PpEBF1* significantly increased the ubiquitination of PpJAZ1 (Supplementary Fig. S5), indicating that PpJAZ1 serves as a ubiquitination substrate of PpEBF1. Additionally, an *in vitro* cell-free protein degradation assay was employed to assess the impact of PpEBF1 on PpJAZ1 protein stability. Overexpression of *PpEBF1* shortened the half-life of PpJAZ1 (Fig. 1I). Taken together, these results demonstrate that PpEBF1 mediates the targeted degradation of PpJAZ1 through the ubiquitin–proteasome pathway, thereby playing a crucial role in the crosstalk regulation between JA and ET signaling. In addition, we observed widespread interactions between PpEBF1 and PpJAZs (Supplementary Fig. S2A), implying that the EBF1-JAZ module might serve broader functional roles in plant development. The regulatory roles of PpEBF1-mediated degradation of other PpJAZ proteins in growth and developmental processes remain to be elucidated.

Given that PpJAZs lack direct transcriptional regulatory activity, they typically function by interacting with co-regulatory factors such as *PpMYC2*. Y2H assay revealed an interaction between PpMYC2 and PpJAZ1 (Fig. 1J), which was further validated through BiFC, LCI, and pull-down assays (Supplementary Fig. S6). Moreover, transient transformation assays in peach leaves and fruits indicated that *PpMYC2* positively regulates peach BS disease resistance (Fig. 1K–N, Supplementary Figs S7 and S8). In plant defense responses, to counteract polygalacturonase (PG) secreted by pathogens, plants express POLYGALACTURONASE-INHIBITING PROTEIN (PGIP), which specifically binds to and inhibits PG activity. This mechanism mitigates PG-mediated degradation of the host cell wall, maintains cellular structural integrity, and effectively prevents pathogen invasion. Previous studies have confirmed that the JA signaling pathway activates *PpPGIP1* expression through the transcription factor PpMYC2, thereby participating in oligosaccharide-induced disease resistance in peach fruits [5]. In this study, yeast one-hybrid (Y1H) assay revealed that PpMYC2 directly bound to the promoter region of the disease resistance-related gene *PpPGIP1* (Fig. 1O, Supplementary Table S1). Dual-luciferase reporter assay (luciferase (LUC)/renilla (REN)) indicated that PpMYC2 significantly activated *PpPGIP1* transcription (Supplementary Fig. S9). Notably, compared to expression of *PpMYC2* alone, co-expression with *PpJAZ1* significantly attenuated the transcriptional activation of downstream *PpPGIP1* by PpMYC2 (Fig. 1P). These findings suggested that PpJAZ1 participates in the regulation of plant disease defense responses by antagonizing PpMYC2 function.

Interestingly, we found that exogenous application of MeJA significantly induced the expression of *PpEBF1* (Supplementary Fig. S10). Moreover, overexpression of *PpMYC2* in leaves and fruits resulted in an upregulation of *PpEBF1* (Supplementary Fig. S11). Analysis of promoter cis-acting elements revealed a typical PpMYC2-binding motif within the *PpEBF1* promoter region (Supplementary Table S2). Electrophoretic mobility shift assay (EMSA) confirmed that PpMYC2 directly bound to the CACATG motif (Fig. 1Q). Meanwhile, LUC/REN assay showed that PpMYC2 significantly activated the transcription of *PpEBF1* (Fig. 1R). Collectively, these findings demonstrate that *PpEBF1* is a direct downstream target gene of PpMYC2. All primer sequences described in this article are shown in (Supplementary Table S3).

In summary, our study reveals that PpEBF1 mediates the ubiquitin-dependent degradation of PpJAZ1. This degradation relieves PpJAZ1’s repression of PpMYC2, leading to PpMYC2-mediated activation of the *PpPGIP1* gene and the initiation of JA-dependent disease defense. Notably, JA-activated PpMYC2 further positively regulates the transcriptional expression of *PpEBF1*, establishing a self-reinforcing positive feedback loop that significantly amplifies JA-mediated plant defense responses (Fig. 1S). We propose that this finely tuned signal amplification mechanism, orchestrated by the PpMYC2–PpEBF1–PpJAZ1 regulatory module, may serve as a molecular basis for plants to achieve efficient resource reallocation and dynamically optimize disease resistance strategies under complex environmental conditions. Collectively, these findings not only systematically elucidate the dynamic post-translational regulatory network of JAZ proteins but also reveal a novel paradigm wherein plants maximize defense efficacy through the crosstalk of hormone signaling networks in response to pathogen stress.

## Data Availability

All data are available within the manuscript and its supporting materials in the figshare (https://doi.org/10.6084/m9.figshare.32103811).
